# An optimized protocol for the extraction and quantification of cytosolic DNA in mammalian cells

**DOI:** 10.1016/j.xpro.2024.102913

**Published:** 2024-02-22

**Authors:** Aminu S. Jahun, Frederic Sorgeloos, Ian G. Goodfellow

**Affiliations:** 1 Division of Virology, Department of Pathology, University of Cambridge, Addenbrooke’s Hospital Level 5, Hills Road, CB2 0QQ Cambridge, UK; 2 Université catholique de Louvain, de Duve Institute, MIPA-VIRO 74-49, 74 Avenue Hippocrate, 1200 Brussels, Belgium

**Keywords:** Cancer, Cell Biology, Cell separation/fractionation, Cell-based Assays, Immunology, Molecular Biology

## Abstract

Leakage of mitochondrial or nuclear DNA into the cytosol can occur following viral infections, radiation damage, and some cancers. Here, we present an optimized protocol for isolating and quantifying cytosolic DNA from mammalian cells. We describe steps for collecting cytosolic fractions from cells, extracting DNA using columns, and quantifying extracted DNA using qPCR. This straightforward protocol can be completed in as little as 5 hours, and allows for the identification of the source of DNA.

For complete details on the use and execution of this protocol, please refer to Jahun et al.^1^

## Highlights

• Detailed steps for cell lysis using mild digitonin buffer
• Fractionation of the lysate to isolate the cytosolic fraction
• Extraction of DNA from the cytosolic fraction using spin columns
• Quantification of the cytosolic DNA using qPCR

## Abstract (editor highlights)

Publisher’s note: Undertaking any experimental protocol requires adherence to local institutional guidelines for laboratory safety and ethics.

## Graphical abstract

**Figure:**
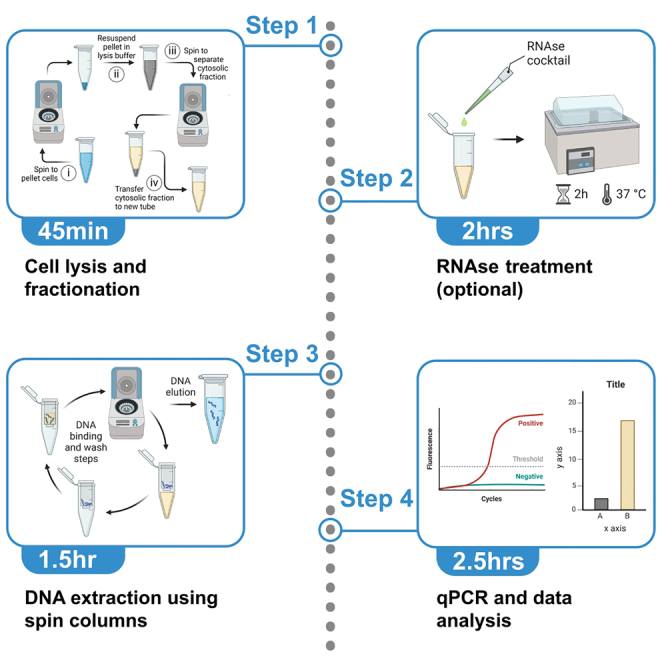

## Before you begin

In eukaryotic cells, host DNA is normally sequestered within the nucleus and mitochondria during interphase. However, leakage and accumulation in the cytosol can occur in conditions such as; (1) Infections with viruses, including Dengue, Chikungunya and noroviruses, where it is thought that lipid recruitment for replication complex formation causes damage to mitochondrial and/or nuclear membranes; (2) Cancer cells, especially those involving defects in DNA repair proteins; (3) DNA damage following exposure to radiation or chemicals such as etoposide; and (4) Cellular defect in certain proteins such as BANF1, TREX1 or DNase II. (For a recent review on leakage of DNA into the cytosol, see Miller et al.^2^).

This protocol uses cell fractionation and column extraction to isolate DNA from the cytosol of cells, and relative quantitative PCR (qPCR) to quantify it relative to mock conditions. As an example, we use cells infected with the mouse norovirus (MNV), which was recently shown to trigger leakage of genomic and mitochondrial DNA into the cytosol.^1^ We used previously published primer pairs targeting *18S* (nuclear-DNA1), *GAPDH* (nuclear-DNA2), the *RNR2-TRNL1 junction* (mtDNA1), the *D-loop region* (mtDNA2), *COX2* (mtDNA3), and *ATP6* (mtDNA4) genes.^3^ It is expected that users will have prior experience with qPCR. For an introduction to qPCR, see Nolan et al.^4^

While this protocol is based on extraction of cytosolic DNA from HEK293T cells infected with MNV, it has also been applied to RAW264.7 cells infected with MNV, as well as HEK293T cells transfected with the MNV NS4 protein.^1^ A similar protocol has also been used in EMCV- or Influenza A virus (IAV)-infected HEK293FT and MEF cells, and HEK293FT cells expressing EMCV 2B or IAV M2 proteins.^5^

Advantages of this protocol over others, such as those involving imaging, include; (1) It is quick (can be completed in as little as 5 hours, with only 3 hours of hands-on work); (2) It is easy to perform (does not require specialized skills or equipment); (3) It avoids the use of phenol/chloroform by employing spin columns; (4) It allows for the identification of the type or source of DNA involved (nuclear, mitochondrial, viral, etc.); and (5) It provides DNA material for further analyses (such as sequencing) or functional assays.

### Institutional permissions

All experiments in this protocol must be performed according to relevant regulatory and safety standards, and after acquiring permissions from the appropriate institutions.

### Preparation: Infection of cells with MNV (Day 0)

**Timing: 18 h**

This section describes infection of HEK293T cells stably expressing mouse CD300lf with the mouse norovirus 1 (MNV1) at an MOI of 0.05 TCID_50_ per cell. Ectopic expression of the mouse CD300lf protein, the entry receptor for MNV, renders HEK293T cells susceptible to infection with the virus. MNV infection triggers leakage of DNA into the cytosol,^1^ making it a suitable model for demonstrating the application of this protocol.

1. Collect and count the cells.

***Note:*** For HEK293T cells, we collect them by incubating in Accutase Cell Detachment solution (BioLegend, 423201) for 5 minutes at 37°C followed by resuspension in complete DMEM (DMEM supplemented with 10% FBS, L-Glutamine, non-essential amino acids, and penicillin/streptomycin). We recommend ensuring there are enough cells for the assay at this point, before going any further. We normally carry out this assay using 2 to 3 replicates per condition, with 4×10^6^ cells per replicate. For the example infection described in this section, we used 3 replicates each for mock and MNV-infected conditions, thus requiring 24×10^6^ cells in total.

2. Centrifuge at 800 × *g* at 4°C for 5 min.
3. Discard the media and resuspend the cells at 4 × 10^6^ per mL.
4. Transfer 1 mL (4 × 10^6^ cells) each into six 1.5 mL tubes (three ‘infected’ and three ‘mock’ tubes).
5. Add appropriate volume of virus at an MOI of 0.05 TCID_50_ per cell, or complete DMEM in the three ‘infected’ or three ‘mock’ tubes, respectively.

***Note:*** We recommend having the virus inoculum between 50‒300 μL, for a final volume of 1.05‒1.3 mL after adding it to the cells. Volumes less than 50 μL may increase technical variability. If a higher volume of virus is required to achieve the desired MOI, we recommend resuspending the cells in a smaller volume so that the final volume is still between 1.05‒1.3 mL.

6. Incubate tubes on an end-to-end rotator at 37°C for 1 h.

***Note:*** We normally use a Labinco L28 fixed speed rotator that has a speed of 10 RPM, but any laboratory end-to-end rotator of around 10–20 RPM should work the same.

7. Centrifuge at 800 × *g* for 5 min at 4°C.
8. Discard the media and resuspend the cells in 1 mL of media.
9. Repeat steps 7–8.
10. Transfer cells into 10 cm dishes, and add 9 mL of pre-warmed media.
11. Incubate at 37°C, 5% CO_2_, for harvesting 18 h post-infection.

## Key resources table

**Table:** 

REAGENT or RESOURCE	SOURCE	IDENTIFIER
**Antibodies**		

Mouse monoclonal anti-GAPDH (1:5,000)	Ambion	Cat#AM4300; RRID: AB_437392
Rabbit polyclonal anti-NS7 (1:500)	Non-commercial	N/A

**Bacterial and virus strains**		

MNV1 (MNV-1.CW1)	Chaudhry et al.^6^	GenBank accession #: DQ285629.1

**Chemicals, peptides, and recombinant proteins**		

Digitonin (5%)	Invitrogen	Cat#BN2006
HEPES (1 M)	Gibco	Cat#15630-056
SybrGreen	Molecular Probes	Cat#S-7563
qPCR core kit SybrGreen no Rox	Eurogentec	Cat#RT-SN73-05NR
ROX passive reference	Eurogentec	Cat#RT-PARE-03
DMSO	Sigma	Cat#D-5879
Betaine	Sigma	Cat#61962
RNase cocktail enzyme mix	Invitrogen	Cat#AM2286
Dulbecco’s modified Eagle’s medium (DMEM)	PAN-Biotech	Cat#P04-03600
Fetal bovine serum (FBS)	Life Science Production (LSP)	Cat#S-001A-HI-BR
L-glutamine	Sigma-Aldrich	Cat#G7513-100ML
Penicillin-Streptomycin	Sigma-Aldrich	Cat#P4333-100ML
MEM non-essential amino acid solution (100×)	Sigma-Aldrich	Cat#M7145-100ML

**Critical commercial assays**		

QIAquick Nucleotide Removal kit	QIAGEN	Cat#28304
QIAamp DNA Mini Kit	QIAGEN	Cat#51304

**Experimental models: Cell lines**		

Human: HEK293T-CD300lf	Jahun et al.^1^	N/A

**Deposited data**		

Uncropped western blots	This paper	Publicly available via Mendeley Data: https://doi.org/10.17632/wpncvmbnty.1

**Experimental models: Cell lines**		

Human: HEK293T-CD300lf	Jahun et al.^1^	N/A

**Oligonucleotides**		

Primers: nuclear-DNA1 (*18S*) 5′-TAGAGGGACAAGTGGCGTTC-3′ 5′-CGCTGAGCCAGTCAGTGT-3′	Aguirre et al.^3^	N/A
Primers: nuclear-DNA2 (*GAPDH*) 5′- CTCTGCTCCTCCTGTTCGAC-3′ 5′- AATCCGTTGACTCCGACCTT-3′	Aguirre et al.^3^	N/A
Primers: mtDNA1 (*RNR2-TRNL1 junction*) 5′- CACCCAAGAACAGGGTTTGT-3′ 5′- TGGCCATGGGTATGTTGTTAA-3′	Aguirre et al.^3^	N/A
Primers: mtDNA2 (*D-loop region*) 5′- CTATCACCCTATTAACCACTCA-3′ 5′- TTCGCCTGTAATATTGAACGTA-3′	Aguirre et al.^3^	N/A
Primers: mtDNA3 (*COX2*) 5′- AATCGAGTAGTACTCCCGATTG-3′ 5′- TTCTAGGACGATGGGCATGAAA-3′	Aguirre et al.^3^	N/A
Primers: mtDNA4 (*ATP6*) 5′- AATCCAAGCCTACGTTTTCACA-3′ 5′- AGTATGAGGAGCGTTATGGAGT-3′	Aguirre et al.^3^	N/A

**Software and algorithms**		

Prism 9.5.0	GraphPad	https://www.graphpad.com/scientific-software/prism/; RRID: SCR_002798
Image Studio Lite 5.2	LI-COR	https://www.licor.com/bio/image-studio-lite/; RRID: SCR_013715
BioRender web tool	BioRender	https://www.biorender.com/; RRID: SCR_018361

**Other**		

ViiA7 real-time PCR system	Thermo Fisher Scientific	https://www.thermofisher.com/uk/en/home/life-science/pcr/real-time-pcr/real-time-pcr-instruments/viia-7-real-time-pcr-system
Centrifuge 5427 R - microcentrifuge	Eppendorf	Cat#5429000065
Labinco L28 Test-tube rotator	Labinco	https://labinco-bv.com/product/labinco-l28-test-tube-rotator/
Eppendorf Xplorer plus automated pipette, single channel, 15–300 μL	Eppendorf	Cat#4861000724

## Materials and equipment

**Table:** Lysis buffer

Reagent	Final concentration	Amount
NaCl (5 M)	150 mM	0.3 mL
HEPES (1 M) pH7.2–7.5	50 mM	0.5 mL
Digitonin (5%)	0.002% (20 μg/mL)	0.04 mL
ddH_2_O	N/A	9.196 mL
**Total**		**10 mL**

Note on storage: Can be stored at 4°C for up to 3 months.

**Table:** 2x qPCR master mix

Reagent	Final concentration (1x)	Amount
ddH_2_O	N/A	3.840 mL
Reaction Buffer (10x)	1x	2.5 mL
MgCl_2_ (50 mM)	2.5 mM	1.25 mL
dNTPs (5 mM)	0.2 mM	1 mL
SYBR Green 1:1,000 in DMSO	1:20,000	1.25 mL
Betaine (5 M)	0.5 M	2.5 mL
Gold Star polymerase (5,000 units/mL)	25 units per mL	0.125 mL
ROX Passive Reference	1:714 (1.4 μL of ROX per mL of master mix)	0.035 mL
**Total**		**12.5 mL**

Note on storage: Can be stored at ‒20°C for up to 3 years. Avoid freeze-thawing more than twice.

***Note on preparing the 2x qPCR mastermix:*** Most of the components of this master mix are part of the qPCR core kit SYBR Green (Eurogentec RT-SN73-05NR), except the SYBR Green reagent (Molecular Probes S-7563), Betaine (Sigma, 61962), and ROX passive reference dye (Eurogentec, RT-PARE-03). While the qPCR core kit comes with SYBR Green, our protocol was optimized using the SYBR Green from Molecular Probes (S-7563).

***Note on preparing 1:1,000 SYBR Green:*** To prepare the 1:1,000 SYBR Green stock, add 20 μL SYBR Green I (Molecular Probes, S-7563) to 20 mL DMSO (Sigma, D-5879), divide this into 1.5 mL aliquots, and keep at ‒20°C (up to 1 year). When preparing the 2x qPCR master mix, it may take up to 1 hour to thaw the 1:1,000 SYBR Green aliquots – while it is thawing, we recommend protecting it from light, such as by covering the tubes in aluminium foil. Once thawed, we normally discard any leftover 1:1,000 SYBR Green reagent.

***Note on preparing 5 M betaine:*** To prepare the 5 M betaine stock, weigh 14.64 *g* of betaine (Sigma, 61962), adjust the volume to 20 mL with ddH_2_O, and dissolve over 60 minutes at room temperature, under gentle agitation. Once it is completely dissolved, adjust the volume to 25 mL with ddH_2_O and divide into 1.5 mL aliquots. Keep at ‒20°C (up to 1 year).

***Alternatives:*** Other commercial SYBR Green or probe-based qPCR master mixes can be used instead, although initial optimization may be required.

## Step-by-step method details

### Cell lysis and fractionation (Day 1)

**Timing: 45 min**

This section involves cell lysis using a digitonin buffer, followed by fractionation to obtain the cytosolic fraction. All steps here are to be carried out on ice or at 4°C.

1. Label 1.5 mL tubes and keep on ice. Pre-chill the centrifuge to 4°C.

***Note:*** These preparatory steps should be carried out 30 minutes before the final incubation is complete. Each condition requires 7–8 tubes per replicate: 1 tube for the lysis, 4 tubes for the fractionation steps, 1 tube for the whole cell sample, 1 tube the cytosolic fraction, and 1 tube for the optional western blot sample. This makes labeling of the tubes tedious and time consuming. While every user will likely develop a labeling strategy that is convenient for them, in our lab we use numbers to indicate each condition and letters to indicate replicates. We add dots or small circles on the tubes for the fractionation steps, with the number of dots indicating the centrifugation step – for example the first tubes to be spun will have 1 dot, the next set of tubes will have 2 dots, etc.

**CRITICAL:** All steps in this section are to be carried out on ice or at 4°C.

2. When the final incubation is complete, take out the cells from the incubator.

***Note:*** The number of cells required per replicate per condition will depend on the cell line used, and may need to be empirically determined. For HEK293T and HeLa cells, we have used between 2‒6×10^6^ cells, and for RAW264.7 cells, we use 4‒6×10^6^ cells.

3. Discard the media, and add 1 mL of ice-cold PBS.
4. Collect the cells by gentle pipetting using a P1000 pipette set at 1,000 μL.

**CRITICAL:** When harvesting cells, avoid the use of cell scrapers if possible. HEK293T cells are usually loosely adherent and can thus be collected by gentle pipetting. For more adherent cell lines such as HeLa cells, we recommend chemical detachment using Trypsin or Accutase.

5. Transfer the cells into 1.5 mL tubes, and place the tubes back on ice.

**CRITICAL:** Avoid freezing the samples prior to fractionation.

6. Centrifuge at 800 × *g*, 4°C, for 5 min.
7. Discard the PBS, and gently resuspend the pellet in 600 μL of lysis buffer.
8. Keep on ice for 10 min.
9. Transfer 400 μL into new tubes as follows:
  a. Mix gently by pipetting up and down.
  b. Transfer 400 μL into new tubes – this is the sample that will be used for the fractionation steps to extract the cytosolic fraction.
  c. Keep the original tubes containing the remaining 200 μL of lysates on ice, for use in steps 15 and 16.
10. Centrifuge the new tubes at 1,000 × *g*, 4°C, for 3 min.
11. Transfer the supernatant into a new tube. Discard the pellet.

***Note:*** Ensure as much supernatant as possible is carried over without disturbing the pellet.

12. Repeat steps 10–11 twice so that the centrifugation at 1,000 × *g* has been carried out 3 times.
13. Centrifuge at 17,000 × *g*, 4°C, for 10 min.

***Note:*** These sequential centrifugation steps separate the cytosolic fraction (the supernatant) from the other components of the cell (the pellet) that includes the nucleus, mitochondria, and other organelles.

14. Transfer the supernatant into a new tube. This is the cytosolic fraction.
15. From the original lysis tubes (step 7), mix gently by pipetting up and down, and transfer 100 μL into new tubes. These are the whole cell lysates.
16. The remaining 100 μL of lysates in the original lysis tubes can either be discarded or used for western blotting.
17. Samples can now be frozen at ‒20°C or used immediately for the next steps.

**Pause point:** Step 17 can be used as a pause point, if needed. Samples can be stored at ‒20°C until required for the next steps, but we recommend avoiding multiple freeze-thaw cycles along the same experiment. While we have not seen any noticeable DNA loss after 1–2 weeks at ‒20°C, we usually carry out all the steps of the protocol sequentially without interruption, since DNA degradation, however minor, is inevitable with multiple freeze thaw cycles.

### RNase treatment

**Timing: 2 h**

This optional part involves degradation of RNA using an RNase cocktail. This can be skipped if samples are only required for qPCR, but not if samples are to be used for later transfection or sequencing. If RNase treatment is not required, skip to step 23.

18. Add 2.5 μL of RNase cocktail into each sample.

***Note:*** The amount of RNase cocktail used here is based on the manufacturer’s recommendation for a typical miniprep, and in our hands is sufficient to reduce viral replication to undetectable levels following transfection of extracted cytosolic DNA from MNV-infected cells into new cells, and to completely degrade ribosomal RNA as visualized on agarose gels. For other applications, the amount of RNase cocktail required may need to be empirically determined.

19. Mix gently.

***Note:*** Steps 18–19 can be integrated into the previous section by adding 2.5 μL of RNase cocktail into new labeled 1.5 mL tubes during the centrifugation in step 13. These tubes are then used for steps 14–15.

20. Incubate at 37°C for 1–2 h.
21. Put tubes back on ice.
22. Samples can now be frozen at ‒20°C or used for the next steps.

**Pause point:** Step 22 can be used as a pause point, if needed. Samples can be stored at ‒20°C until required for the next steps, but we recommend avoiding multiple freeze-thaw cycles along the same experiment.

### Extraction of cytosolic DNA

**Timing: 45 min**

Here, DNA from the cytosolic fraction is extracted using spin columns. This section uses the QIAquick Nucleotide Removal Kit (Qiagen), and the protocol is adapted from the manufacturer’s manual.

23. Transfer 4 mL of PNI buffer into 15 mL tubes.
24. Transfer the cytosolic fractions into the tubes in step 23 above, and mix well by inverting the tubes several times.
25. Transfer the samples into the columns provided, 650 μL at a time, each time discarding the flow through after centrifuging at maximum speed (>12,000 × *g*) for 30 s at room temperature.

***Note:*** Use of a vacuum manifold in place of a centrifuge for steps 25–28 saves a considerable amount of time.

26. Add 500 μL PE buffer into each column and spin at maximum speed (>12,000 × *g*) for 30 s.
27. Discard the flow through.
28. Repeat steps 26–27.
29. Replace the receiving tube with a new one and spin at maximum speed (>12,000 × *g*) for 1 min.
30. Transfer the columns unto labeled 1.5 mL tubes.
31. Add 100 μL of elution buffer into the columns.
32. Keep at room temperature on the bench for 1–5 min.
33. Spin at maximum speed (>12,000 × *g*) for 1 min.
34. Discard the columns and keep the tubes containing the eluates on ice.

***Note:*** Concentrations of the DNA samples can be measured using a NanoDrop spectrophotometer or a Qubit fluorometer at this step.

35. Samples can now be frozen at ‒20°C or used for the next steps.

**Pause point:** Step 35 can be used as a pause point, if needed. Samples can be stored at ‒20°C until required for the next steps, but it is best to avoid multiple freeze-thaw cycles along the same experiment.

### Extraction of whole cell DNA

**Timing: 30 min**

Here, DNA from the whole cell fraction is extracted using spin columns. This section uses the QIAamp DNA mini kit (QIAGEN), and the protocol is adapted from the manufacturer’s manual.

36. Adjust the volume of the whole cell samples with PBS to obtain a total volume of 200 μL.
37. Add 20 μL of proteinase K and mix gently by pipetting up and down ten times.
38. Add 200 μL of buffer AL.
39. Vortex briefly to mix, and spin briefly to collect the samples at the bottom of the tube.
40. Incubate on a heat block at 56°C for 10 min.

***Note:*** A water bath can be used in place of a heat block.

41. Once incubation is completed, add 200 μL of 100% ethanol.
42. Vortex briefly to mix, and spin briefly to collect the samples at the bottom of the tube.
43. Transfer the samples into the columns provided and spin at maximum speed (>12,000 × *g*) for 30 s.
44. Discard the flow through.
45. Add 500 μL of AW1 buffer and spin at maximum speed (>12,000 × *g*) for 30 s.
46. Discard the flow through.
47. Add 500 μL of AW2 buffer and spin at maximum speed (>12,000 × *g*) for 30 s.
48. Discard the flow through.
49. Replace the receiving tube with a new one and spin at maximum speed (>12,000 × *g*) for 1 min.
50. Transfer the columns into new 1.5 mL tubes, and add 100 μL of elution buffer.

***Optional:*** Keep at room temperature on the bench for 1‒5 minutes.

51. Spin at maximum speed (>12,000 × *g*) for 1 min.
52. Discard the columns and keep the tubes containing the eluates on ice.

***Note:*** Concentrations of the DNA samples can be measured using a NanoDrop spectrophotometer or a Qubit fluorometer at this step.

53. Samples can now be frozen at ‒20°C or used for the next steps.

**Pause point:** Step 53 can be used as a pause point, if needed. Samples can be stored at ‒20°C until required for the next steps, but we recommend avoiding multiple freeze-thaw cycles along the same experiment.

### Quantitative PCR

**Timing: 2 h 15 min**

This section involves diluting the DNA samples, setting up the qPCR plates and running the samples in a qPCR machine.

54. All work should be carried out on ice.
55. Determine the number of reactions to be run and prepare the master mix as shown on Table 1.

**Table 1:** PCR reaction master mix

Reagent	Amount per reaction
ddH_2_O	7 μL
Forward primer (10 pmol/μL)	0.25 μL
Reverse primer (10 pmol/μL)	0.25 μL
2x master mix	12.5 μL
**Total**	**20 μL**

***Note:*** We run each sample in 3 technical replicates (i.e. 3 technical replicates for each biological replicate), and include 2 or more non-template controls per primer pair. Also, remember to add one extra reaction for every 10 samples to account for pipetting error. For example, if you have 6 samples of cytosolic DNA and 6 samples of whole cell DNA, you will need to prepare enough master mix for 42 reactions for every primer pair (36 reactions for 12 samples in triplicates, 2 reactions for non-template controls, and 4 for pipetting error).

56. Keep the master mix on ice.
57. Dilute the DNA samples from steps 35 and 53 1:10 in ddH_2_O.
58. Aliquot 20 μL of appropriate master mix into each well of a qPCR plate.

***Note:*** This is easier done using an electronic pipette/dispenser.

59. Add 5 μL of the diluted DNA to produce 3 technical replicates, or water (non-template control) into each appropriate well.

***Note:*** Using a multichannel pipette in this step reduces hands-on time and reduces variability between technical replicates. We usually distribute each sample from step 57 into 3 tubes of 0.2 mL strip tubes and then use multichannel pipettes to transfer 5 μL into appropriate wells of the qPCR plate.

60. Seal plate with clear adhesive and spin briefly in the table-top centrifuge.
61. Run samples on a qPCR machine using the cycling conditions indicated in Table 2.

**Table 2:** qPCR cycling conditions

Steps	Temperature	Time	Cycles
Initial Denaturation	55°C	2 min	1 cycle
	95°C	10 min	
Denaturation	95°C	15 s	40 cycles
Annealing and extension	60°C	60 s

**Pause point:** Step 61 can be used as a pause point, if needed.

### Data analysis

**Timing: 15 min**

Here, we calculate the fold change compared to mock, using the Livak (2^ΔΔCt^) method,^7^ by normalizing the cytosolic DNA in each sample with its corresponding whole cell DNA, and then with the mock-infected samples.

62. Obtain the Ct (cycle threshold) values from the qPCR machine, and calculate the average Ct values of the technical replicates.
63. Calculate the delta-Ct by subtracting the average Ct values of each cytosolic fraction from its corresponding whole cell fraction.
64. Calculated the average delta-Ct of the mock samples.
65. Calculate the delta-delta-Ct by subtracting the average delta-Ct of the mock from the delta-Ct values of each sample.
66. Calculate the exponential function of the delta-delta Ct values with base 2. These results represent the fold change compared to mock.

**Table 3:** Example of raw data obtained following qPCR using the nuclear-DNA1 (18S) primer pair

		Whole cell (WC) DNA Ct values	Cytosolic (cyt) DNA Ct values	Average Ct of WC DNA	Average Ct of cyt DNA	ΔCt	Average ΔCt of mock	ΔΔCt	2^ΔΔCt^
rep 1	mock	17.386	27.906	17.390	27.939	−10.549	−10.616	0.067	1.048
		17.363	28.044						
		17.422	27.869						
rep 2	mock	17.383	27.962	17.387	28.034	−10.646		−0.030	0.980
		17.348	28.065						
		17.431	28.074						
rep 3	mock	17.533	28.109	17.444	28.098	−10.654		−0.037	0.974
		17.419	28.165						
		17.378	28.019						
rep 1	MNV1	16.982	24.102	16.909	24.104	−7.195		3.421	10.714
		16.890	24.155						
		16.855	24.055						
rep 2	MNV1	16.957	24.111	16.868	24.146	−7.278		3.339	10.116
		16.801	24.187						
		16.847	24.141						
rep 3	MNV1	16.957	24.105	16.990	24.144	−7.154		3.462	11.021
		16.989	24.181						
		17.022	24.145						

**Figure 1:**
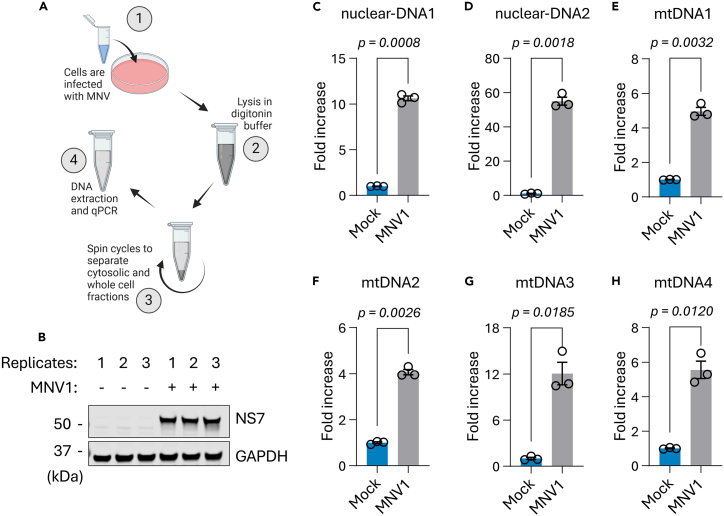
Representative results showing leakage of DNA into the cytosol in MNV1-infected cells (A) Schematic representation of the workflow. (B‒H) HEK293T cells stably expressing the mouse CD300lf receptor were infected with MNV1 in 3 replicates at an MOI of 0.05 TCID50/cell. Cells were harvested 18 h post-infection and cytosolic DNA was assessed relative to mock-infected cells. Whole cell lysates were used for western blotting for MNV1 NS7 and GAPDH, to show virus infection (B). Panels (C) to (H) show levels of indicated cytosolic DNA in infected cells normalized to individual whole cell DNA, relative to mock-infected conditions. Error bars represent standard error of mean. nuclear-DNA1 = *18S*, nuclear-DNA2 = *GAPDH*, mtDNA1 = *RNR2-TRNL1 junction*, mtDNA2 = *D-loop region*, mtDNA3 = *COX2*, mtDNA4 = *ATP6*.

This is the same data plotted in Figure 1C.

***Note:***Table 3 shows example raw data and the data analysis steps. In this example, the cells were either mock-infected or infected with MNV1 in 3 replicates each, and each of these was in turn ran as 3 technical replicates.

## Expected outcomes

Figure 1 shows a typical result using this protocol on cells infected with an acute strain of the mouse norovirus (MNV1). The cells were infected with MNV1 at a low multiplicity of infection over 18 h, followed by fractionation, extraction of DNA, and relative quantification by qPCR (Figure 1A). This was carried out using 3 replicates each for the mock-infected and infected cells, with a total of 6 10-cm dishes. Infection was successful as indicated by the detection of the viral NS7 protein in all 3 infected replicates, but not in the mock-infected cells (Figure 1B). Table 3 shows the raw data for Figure 1C, and the data analysis steps. MNV1 leads to release of genomic and mitochondrial DNA (Figures 1C–1H) that go on to induce an innate immune response.^1^

## Limitations

According to the manufacturer, optimal binding for the columns used in this protocol is seen for DNA fragments between 40 to 10,000 base pairs, which is a wide enough range. Still, this can potentially introduce a bias for DNA fragments outside of this range.

The times given in this paper assumes one is working with 6 samples or less at a time, and any increase in the number of samples beyond that also increases the time it takes to complete. Thus, one limitation of this protocol is that the number of samples that can be processed at the same time is limited, and it is not as high throughput as protocols that involve imaging. Still, some parts of the protocol, including the DNA extraction steps (steps 23–53), are amenable to automation.

Additionally, while using whole cell DNA in place of a housekeeping gene for the relative quantification of cytosolic DNA controls for any variation in total cellular DNA between replicates and experimental conditions, such as that from changes in total mitochondrial numbers, we assume that any changes in the total cellular DNA does not affect the proportion of DNA leaking into the cytosol. Conditions that lead to asymmetric changes in the total DNA will therefore give spurious results.

Other limitations of qPCR also apply to this protocol, and have been discussed by Nolan et al.^4^ and by Smith et al.^8^

## Troubleshooting

### Problem 1

Excessive background.

### Potential solutions

• Ensure that where indicated, all the steps are carried out on ice or at 4°C.
• Avoid using cell scrapers to detach the cells (step 4).
• Avoid freezing the samples prior to fractionation (step 14).
• Run the experiment alongside a positive control, such as cells treated with Thapsigargin.

### Problem 2

High variability between technical replicates.

### Potential solutions

• Ensure good pipetting technique.
• Ensure that where indicated, all the steps are carried out on ice or at 4°C.
• In step 57, make sure samples are sufficiently mixed after dilution.
• Use an automated pipette/dispenser when adding the qPCR master mix into the qPCR plate (step 58) and a multichannel pipette when adding the samples into the qPCR plate (step 59).

### Problem 3

No amplification in the whole cell DNA samples.

### Potential solutions

• Ensure that all components of the qPCR master mix have been added.
• Make sure that the primers used are validated and are used at the correct concentrations (step 55).
• Check that the samples (from step 53) contain DNA, for example by running them on an agarose gel.

### Problem 4

Multiple peaks on the melt curve.

### Potential solutions

• Make sure that the primers used are validated and are used at the correct concentrations (step 55).

### Problem 5

Amplification seen in the non-template control.

### Potential solutions

• If these have the same peak on the melt curve as the samples containing DNA, it may indicate contamination. In this case, repeat the qPCR while replacing the master mix, primers and ddH_2_O, one at a time to determine the source of contamination.
• If the peaks on the melt curve are different from those for the samples containing DNA, it may indicate formation of primer dimers. In this case, we recommend revalidating the primers or designing new ones.

***Note:*** For general troubleshooting of qPCR, see Nolan et al.^4^ and Raso et al.^9^

## Resource availability

### Lead contact

Further information and requests for resources and reagents should be directed to and will be fulfilled by the lead contact, Ian G. Goodfellow (ig299@cam.ac.uk).

### Technical contact

Technical questions about the protocol should be directed to and will be answered by the technical contact, Aminu S. Jahun (asj40@cam.ac.uk).

### Materials availability

No new materials or reagents were generated for this work.

### Data and code availability

Uncropped western blots are publicly available via Mendeley Data: https://doi.org/10.17632/wpncvmbnty.1. Otherwise all the data generated or analyzed during this study are included in the manuscript. No new code was generated in this study.
