# An optimized protocol for the extraction and quantification of cytosolic DNA in mammalian cells

**DOI:** 10.1016/j.xpro.2026.104541

**Published:** 2026-04-22

**Authors:** Aminu S. Jahun, Frederic Sorgeloos, Ian G. Goodfellow

## Main text

(STAR Protocols *5*, 102913; March 15, 2024)

Due to author oversight, an error was introduced in the “materials and equipment” section during the preparation of this manuscript. In the lysis buffer table, the amount of 5% digitonin stock should be “0.004 mL” instead of “0.04 mL.” The authors apologize for this error and regret any confusion.

